# Cross-Talk Between Inflammation and Fibroblast Growth Factor 10 During Organogenesis and Pathogenesis: Lessons Learnt From the Lung and Other Organs

**DOI:** 10.3389/fcell.2021.656883

**Published:** 2021-05-31

**Authors:** Manuela Marega, Chengshui Chen, Saverio Bellusci

**Affiliations:** ^1^Key Laboratory of Interventional Pulmonology of Zhejiang Province, Department of Pulmonary and Critical Care Medicine, First Affiliated Hospital of Wenzhou Medical University, Wenzhou, China; ^2^Member of the German Center for Lung Research (DZL), Department of Pulmonary and Critical Care Medicine and Infectious Diseases, Cardio-Pulmonary Institute (CPI), Universities of Giessen and Marburg Lung Center (UGMLC), Justus Liebig University Giessen, Giessen, Germany

**Keywords:** inflammation, alveolar epithelial type 2 cell, lipofibroblast, immune cells, stromal niche, Fgf10

## Abstract

The adult human lung is constantly exposed to irritants like particulate matter, toxic chemical compounds, and biological agents (bacteria and viruses) present in the external environment. During breathing, these irritants travel through the bronchi and bronchioles to reach the deeper lung containing the alveoli, which constitute the minimal functional respiratory units. The local biological responses in the alveoli that follow introduction of irritants need to be tightly controlled in order to prevent a massive inflammatory response leading to loss of respiratory function. Cells, cytokines, chemokines and growth factors intervene collectively to re-establish tissue homeostasis, fight the aggression and replace the apoptotic/necrotic cells with healthy cells through proliferation and/or differentiation. Among the important growth factors at play during inflammation, members of the fibroblast growth factor (Fgf) family regulate the repair process. Fgf10 is known to be a key factor for organ morphogenesis and disease. Inflammation is influenced by Fgf10 but can also impact Fgf10 expression *per se*. Unfortunately, the connection between Fgf10 and inflammation in organogenesis and disease remains unclear. The aim of this review is to highlight the reported players between Fgf10 and inflammation with a focus on the lung and to propose new avenues of research.

## Introduction

In the past few years, the plasticity and dynamics of the immune cells in different organs and during development, homeostasis and repair after injury have become the focus of research. In particular, macrophages have been shown to be more than phagocytic cells and to display different behaviors based on the specific tissues where they are localized. Notably, many studies have demonstrated the heterogeneity of the macrophage subpopulations in different organs, such as the brain and lung. We propose that in the lung, alveolar epithelial cells and associated stromal cells called lipofibroblasts (LIFs) are the main interlocutors of the immune cells. While LIFs are not yet well characterized, alveolar epithelial cells are known to display immune characteristics (e.g., expression of surface markers which allow their interaction with immune cells). Fibroblasts growth factors (Fgfs) are involved in many diseases with inflammatory features, suggesting a strong connection with the immune system. Among them, Fgf10 represents a promising molecule, due to its important role during lung morphogenesis and in the repair process after injury. It has been reported how different levels of *Fgf10* impacts the immune cell populations present in the lung during disease. Inflammation mediators, like NF-κB, could modify the expression level of *Fgf10*. Immune cells could express Fgf10 which acts in a paracrine and/or autocrine fashion. It remains to be clarified which immune cells are involved during development, homeostasis and repair, as well as the molecules at play, in terms of cytokines, chemokines or alarmins. In this review, we will explore what is known about the relationship between Fgf10 and inflammation and the possible approaches that we can develop to better understand and define the cross-talk between these two players. In particular, further studies are necessary to better characterize the role of Fgf10 in the context of inflammation, not only intended as a defense mechanism against aggression, but in the context of organogenesis and repair after injury.

## FGF10 Is Critical During Organogenesis

Fgf10 binds specifically to the Fgf receptor 2b (Fgfr2b), leading to the autophosphorylation of this tyrosine kinase receptor and the subsequent activation of a signaling cascade in the target cells. In the lung, *Fgf10* was first detected in the splanchnic mesoderm surrounding the foregut around E9.5 when the primary lung buds start to emerge (Yuan et al., 2018). From loss of function studies in mice, the importance of *Fgf10* in lung formation appears clear: the newborn mice die at birth due to a failure of lung formation. The primary buds corresponding to the main bronchi form but fail to elongate (Min et al., 1998). Strikingly, the trachea forms but displays abnormal patterning of the cartilage rings (Sala et al., 2011).

Other visceral organs including the pancreas, some specific portions of the gut, as well as ectoderm-derived organs such as the limb, mammary gland, and salivary and lacrimal glands are also impaired (Bellusci et al., 1997; Sekine et al., 1999; Itoh, 2016; Zepp and Morrisey, 2019). The last step of lung development is called alveologenesis, leading to the formation of the alveoli (Branchfield et al., 2015; Chao et al., 2016). In mice, this developmental phase starts shortly after birth [from postnatal day (P)5 through P36]. The alveolus is the basic functional structure containing several different cell types important for gas exchange, homeostasis and repair after injury. The role of Fgf10/Fgfr2b signaling in the alveolar epithelial progenitors giving rise to the alveolar type 1 and type 2 cells (AT1 and AT2, respectively) has been studied mostly from partial loss or gain of function approaches and it is still elusive (Chao et al., 2016). In the context of hyperoxia exposure to mouse pups, which models a human disease called bronchopulmonary dysplasia (BPD), decreased Fgf10 levels lead to premature death associated with profound alveolar defects (quantified by morphometry analysis for parameters such as mean linear intercept) and impaired AT2 differentiation and decreased surfactant production (Chao et al., 2017). Moreover, Kalymbetova et al. (2018) have proposed an active role for resident macrophages in pathological lung development, using a similar mouse model of BPD (85% oxygen). They identified a novel population of immune cells, named population 3 (Pop3), characterized by high expression of the major histocompatibility complex II (MHCII). Because of this high expression, the authors suggested that alveolar macrophages (AMs) can transdifferentiate into Pop3 after exposure of the animals to hyperoxia. At the same time, it was possible to observe arrested alveolar development, strongly suggesting a causative role in arrested lung development for this new macrophage population (Kalymbetova et al., 2018).

## The Immune Cells in the Lung Arise From Different Sources

In the adult lung, the main immune cell type present is the macrophage. Lung macrophages belong to two different subgroups: alveolar macrophages and interstitial macrophages (IMs); the role of each group is still poorly understood, and research focuses mostly on AMs, due to their localization and their abundance. In the lung, AMs are localized in the lumen of the alveolus and are in direct contact with the epithelium. Their main function is phagocytosis of surfactant and irritants. On the other side, the IMs represent 30–40% of the total macrophages, and their role is primarily connected to tissue remodeling and homeostasis, as well as to antigen presentation (Evren et al., 2020).

### The Origin of the Immune Cells Present in the Lung Has Been Clarified

During fetal life, the lung is populated by fetal lung myeloid populations that include fetal macrophages and fetal liver-derived monocytes (De Kleer et al., 2014; van de Laar et al., 2016). Shortly after birth, these immune cells mature into AMs, which are characterized by long-term survival. Throughout life, the population of AMs is maintained via their local proliferation and, therefore, does not normally require the recruitment of the circulating monocytes from the blood. AMs protect against viruses, bacteria, pollution and smoke. The exposure to these irritants, however, leads to a partial substitution of the local AMs with circulating monocytes, which then eventually become resident AMs themselves (Gordon and Plüddemann, 2017; Evren et al., 2020). Moreover, in the past, the proposed role of these cells was defined by the tissue or organ where they localized, with the assumption that all the macrophages originate from circulating monocytes derived from the bone marrow (Takahashi et al., 1989; Takeya and Takahashi, 1992). Over the years, this concept has drastically changed as the macrophages have been reported to originate from three independent sources: the previously mentioned bone marrow, but also the fetal yolk sac and the fetal liver (Tan and Krasnow, 2016). This raises the possibility that, depending on their origin, the macrophages will react differently to their microenvironment. The developmental origin of AM and IM was recently addressed through a combination of immunofluorescence for specific markers, genetic lineage tracing, and parabiotic studies (Tan and Krasnow, 2016).

### Three Different Macrophage Waves Were Described in the Mouse Lung

The macrophage cells constituting each wave are different but share nonetheless phagocytic abilities and antigen presentation capacity. The first wave gives rise to embryonic F4/80^Pos^ lineage macrophages and arises from the yolk sac. These cells spread in the lung interstitium, and during the first week of life, they change localization, moving to the perivascular and peripheral region of the lung. The second wave originates from the fetal liver, and the corresponding macrophages are characterized by the presence of Galectin 3 (aka Mac-2), a carbohydrate-binding protein marker present on their surface. These cells also spread in the interstitial space. During the first week postnatally, they enter in the alveoli as their final destination and display self-renewal capacity to maintain the cell pool size (Röszer, 2018). The third wave comes from circulating monocytes and leads to the formation of the mature interstitial macrophages. These cells replace the embryonic interstitial version, and they are eventually replaced when needed from circulating progenitors (Tan and Krasnow, 2016). Notably, the tissue microenvironment may be more important than the site of origin of the macrophages. A recent study shows that macrophages that arise from different sources can replace the vacant alveolar niche in what some authors have termed “niche competition model” (Guilliams and Scott, 2017). For example, yolk sac macrophages, fetal monocytes, and bone marrow monocytes are all capable of replacing AMs (Evren et al., 2020).

The origin of the interstitial macrophages present in the lung adds an additional level of understanding to the role of these cells. While the role of alveolar and canonical interstitial macrophages has been investigated, the presence of additional subpopulations requires further research for the in-depth characterization of these cellular pools of interstitial cells.

### IM Represent a Minor Population Residing in the Parenchyma

Recently, an interesting work was published describing macrophages and their subpopulations in different tissues, including the lung (Chakarov et al., 2019). The authors identified two, not three, as previously described (Tan and Krasnow, 2016), subpopulations of resident macrophages, which derived from circulating monocytes and which displayed unique signatures based on their localization in different tissues, such as the heart, lung, skin, and in fat. They analyzed further the IMs in murine lung, with the rationale that AMs are the major embryonically-derived population and that IM represent a minor population residing in the parenchyma. IM are defined as Lyve^hi^MHCII^low^CX3CR1^low^ and Lyve^low^MCHII^hi^CX3CR1^hi^ based on the expression levels of these surface markers (Chakarov et al., 2019).

IM Lyve^hi^MHCII^low^CX3CR1^low^ and Lyve^low^MCHII ^hi^CX3CR1^hi^ are characterized by distinctive gene profiles, phenotypes and functions. Focusing on the murine lung, the depletion of one of these two populations leads to the exacerbation of induced fibrosis, demonstrating the specific and relevant role of these cells in the inflammatory process taking place during fibrosis formation (Chakarov et al., 2019). Following activation by the inflammatory process, these dynamic cells could represent a source of Fgf10 and thus drive the repair process with their own plasticity. Therefore, further clarification is needed on the role of these respective immune populations in the lung in order to devise strategies to allow functional recovery of the lung following exposure to toxic biological or chemical agents. Different origins may be associated with different functions, and due to the localization of the embryonic F4/80^Pos^ macrophages, it is easy to speculate a role for these cells in stem cell renewal, combined with growth factor secretion.

## Inflammation and Extracellular Matrix Remodeling: Impact on FGF10 Signaling

Cleavage of heparan sulfate proteoglycans (HSPGs) during extracellular matrix remodeling (ECM) remodeling is a feature of inflammation as the leukocytes reach the site of injury. It has been reported that Fgf10 binds to HSPG, which can regulate Fgf10 activity (Watson and Francavilla, 2018). Inflammation could therefore release Fgf10 from the ECM and make it available for repair. Therefore, the changes in the microenvironment likely play a fundamental role in the modulation of Fgf10 activity. However, though it remains unclear how Fgf10 impacts the inflammatory process, ECM remodeling could represent another point of control and modulation of its activity, especially from the therapeutic point of view. In the lung, ECM could act as a chemoattractant where degraded ECM fragments mimic the effect of cytokines. Of note, elastin itself functions as a chemotactic factor for recruiting monocytes (Sorokin, 2010). Other evidence connects hyaluronic fragments with lung injury: the length of the chain determines the pro- or anti- inflammatory effect. Hyaluronic fragments originate from ECM after matrix metalloproteases digestion. They interact with the Toll like receptor 2/4 (Tlr2/4), whose expression can be inhibited by Fgf10 via Hmgb1 [see section “Lipopolysaccharides (LPS) Are Known to be a Potent Activator of the Innate Immune System and Have Been Used in the Context of *in vitro* Lung Explant Culture”].

## Inflammation and FGF10 Signaling: Lessons Learnt From the Mammary Gland

Intriguingly, the development of the mammary gland is also regulated by Fgf10/Fgfr2b signaling: *Fgf10* deficiency leads to impaired mammary gland formation in mice (as well as the lung, as previously mentioned) (Mailleux et al., 2002). *Fgf10* KO impairs the sprouting of the mammary epithelium into the adjacent fat pad. In addition, the fat pad itself is poorly developed in these KO mice, raising the question whether the defective sprouting is due to the mutant fat pad *per se* (Howlin et al., 2006).

### *Colony-Stimulating Factor 1* (*Csf-1*) Deficient Mice Display Delay in Fat Pad Development

Comparable to these results, *colony-stimulating factor 1* (*Csf-1*) deficient mice display an initial delay in fat pad development, but later, the fat pad resumes its growth and reaches a normal size (Gyorki et al., 2009). This last finding suggests that other mechanisms could be activated to compensate for the lack of *Csf-1* to allow fat pad development to resume; one of the possible candidates is the Fgf10/Fgfr2b axis. Studies in mice revealed that Csf-1/Csf-1 receptor signaling is an important chemotactic signaling pathway for the attraction of macrophages in different tissues. In the lung, the airway epithelium secretes Csf-1 thereby regulating the presence of macrophages and dendritic cells, especially in presence of allergens or irritants (Plaks et al., 2015). Tissue-resident macrophages are localized at distinct regions within the mammary gland and have been implicated in various stages of mammary gland development. Recent studies have also identified distinct myeloid cell populations, including Cd11c^Pos^ antigen presenting cells and Csf-1r^Pos^ macrophages that are localized in close association with ductal epithelial cells in mammary glands (Plaks et al., 2015; Stewart et al., 2019). Mammary gland analysis from *Csf-1* deficient mice demonstrated reduced ductal elongation and branching.

### Fgf10/Fgfr2b Signaling Displays Multiple Roles in Mammary Gland Development and Homeostasis

*Fgf10’s* initial expression in the somite controls the migration of surface ectoderm cells to form the mammary placodes as indicated by the observation that *Fgf10* null fail to form four of the five mammary placodes (Veltmaat et al., 2006). The unique placode still present in the *Fgf10* KO embryos reaches the bud stage but fails to elongate and ramify into the improperly differentiated mammary fat pad. Transplantation studies of mutant mammary bud epithelium into a cleared fat pad in the adult mice indicated that the mutant epithelium is perfectly capable to form a mature ramified structure when placed in a permissive environment (Veltmaat et al., 2006) [reviewed in Rivetti et al. (2020)]. Additionally, Fgfr2b ligands are also crucial in post-natal mammary gland development. *Fgfr2b* is highly expressed in mature mice, when puberty starts, and is needed to complete the maturation of the mammary gland with the development of the terminal end buds (TEBs) (Parsa et al., 2008). The mammary gland is composed of ducts, which are made of luminal epithelial cells surrounded by basal/myoepithelial cells. In mice, the TEBs appear as dilated structures at the duct tips around the first month postnatally. Mammary progenitors for both the luminal and basal/myoepithelial lineages are present in the TEBs. Transient inhibition of Fgfr2b signaling leads to the disappearance of the TEBs. However, the TEBs are only temporarily impacted as they reappear once Fgfr2b signaling is restored. It was demonstrated that luminal epithelial cell proliferation and survival is under control of Fgf10/Fgfr2b signaling, thereby allowing the formation and maintenance of the TEBs (Parsa et al., 2008). Interestingly, inactivation of *Csf-1* and *Fgf10* leads to comparable phenotypic alterations during mammary gland formation, suggesting a possible interaction between these two players in organogenesis.

### Genetic Impairment of Macrophage Formation in Mice Leads to Alteration in the Terminal End Buds

As previously mentioned, genetic impairment of macrophage formation in mice leads to alteration in the terminal end buds, pointing out the importance of macrophages in the regulation of mammary gland branching (Gouon-Evans et al., 2000). Recently, a chemokine receptor called atypical chemokine receptor 2 (Ackr2) has been reported to be fundamental in the macrophage control of this process (Wilson et al., 2017). Ackr2 controls macrophage recruitment in the mammary gland (Wilson et al., 2020), and in concert with Ccr1 and its ligand Ccl7, regulates the number of macrophages present in the mammary gland, thereby impacting the process of mammary epithelial morphogenesis during puberty. In absence of Ccr1, macrophages cannot be stimulated by Ccl7; missing its receptor, Ccl7 is not able to guide the macrophages to the ductal epithelium, resulting in reduced presence of the immune cells. Consequently, the ductal epithelium is not stimulated by the macrophages resulting in delayed branching (Wilson et al., 2017). *Ackr2* null mice display increased levels of chemokines, which are attractants for monocytes and macrophages. This leads to an increased number of these cells in the mammary gland, which is associated with acceleration of the branching process. Ackr2’s effect is likely indirect and the identification of *Ccr1* as a main player adds a new layer in our understanding of the relationship between branching and macrophage dynamics (Wilson et al., 2017).

### The Relationship Between Csf-1 and Fgf10 Is Potentially Relevant for Many Diseases Linked to Developmental Defects

In the future, it will be interesting to determine whether the function assumed by macrophages and their impact on Fgf10 signaling observed in mammary gland could also be occurring in the developing lung. We also propose that these macrophages exhibit additional tissue-specific behaviors. For example, in the lung, AMs could regulate surfactant expression by AT2 cells; while it is already known that Csf-1 deficiency impacts the alveolar macrophage population in the lung of young mice: the Csf-1^op/op^ (corresponding to a complete KO) mice have reduced AMs in the broncho-alveolar lavage compared to the control mice (Shibata et al., 2001); this difference is compensated during aging by higher expression of other molecules, like interleukin 3 (Il3) and matrix metalloproteinases (Mmps). Il3 expression may be the main compensatory effect, but because it is associated with Mmp-2, Mmp-9, and Mmp-12 expression, it could cause structural damage to the lung. Supporting this possibility *Csf-1* null mice developed emphysema at later stages without displaying any sign of inflammation (Shibata et al., 2001).

## Inflammation May Alter FGF10 Expression (And Vice Versa) in Broncho-Pulmonary Dysplasia

### The Inflammation Process Results From Unbalanced Organ Homeostasis and Is Initiated by Acute Injury, Chronic Damage and/or Infections

An excessive inflammatory response causes damage to the organs instead of repairing them. Bronchopulmonary dysplasia (BPD) is a chronic lung disease characterized by airway obstruction and defective gas exchange that affects preterm children. BPD is associated with a high risk of developing respiratory infections, right heart failure, and death during the first year of life (Lignelli et al., 2019). The definition of BPD has evolved, due to the development of new treatments, in particular oxygen ventilation. Currently, the lung structural hallmarks of BPD are fewer and larger alveoli. Therefore, aborted alveologenesis, the last phase of lung development, is an important characteristic of BPD (Lignelli et al., 2019). BPD pathogenesis is still unclear. Many factors, both cellular and molecular, could be involved in the early stage of the disease leading to impaired lung development. Infection and the associated inflammation during pregnancy and during the perinatal time increase the risk of developing BPD and negatively impact lung development. From a mechanistic point of view, studies have focused on FGF10 because of its importance throughout lung development. Interestingly, *FGF10* expression is reduced in BPD patients, suggesting that this growth factor is involved in BPD pathogenesis together with inflammation (Chao et al., 2017).

### A Mouse Model of BPD Allows Studying Mechanistically the Significance of Reduction in *Fgf10* Expression

Contrary to humans, which are born normally with lungs at the alveolar stage, mice are born with lungs at the saccular stage. From a respiratory point of view, newborn mice therefore display an immature lung showing significant similarities with the lungs of prematurely born babies which are at high risk for developing BPD. When newborn mice are subjected to hyperoxia exposure (85% O_2_ for 8 days), they exhibit an arrest in lung development and defects in alveolar development, similar to what is observed in human babies with BPD. These mice do survive but show permanent impairment in alveoli formation and therefore reduced respiratory function, among many other defects (Chao et al., 2017, 2019).

Interestingly, the exposure of *Fgf10* heterozygous newborn mice (with one copy of the *Fgf10* gene deleted) to hyperoxia injury causes the death of the mutant animals within 8 days (Chao et al., 2017). Analysis of the whole lung at E18.5 in wild type (WT), *Fgf10*^+/–^ (50% *Fgf10* expression), and *Fgf10*^*LacZ/*–^ (20–30% *Fgf10* expression) hypomorph mice by microarray revealed a dysregulation at many levels: genes that belong to the epithelium, nerve, immune system, muscle and ECM showed an up or down-regulation that correlated with the expression level of *Fgf10*. Analysis of the WT and *Fgf10* heterozygous isolated AT2s at postnatal day 3 in normoxia and hyperoxia by microarrays revealed a further strong dysregulation in genes involved in inflammation: for example, autoimmune diseases (systemic lupus erythematosus, auto-immune thyroid disease, and graft - versus- host disease) and antigen processing presentation. Therefore, these results suggest that AT2 cells display different immune status in *Fgf10* heterozygous compared to wild type control lungs, which could, in turn, differentially modulate the inflammatory response (Prince et al., 2001; Benjamin et al., 2007). Interestingly, as altered immune gene signature of the AT2 cells was observed in the *Fgf10* heterozygous neonate mice in the context of hyperoxia (Chao et al., 2017), it is likely that decreased Fgf10 expression is causative for the effects on inflammation.

### AT2 Cells Can Impact Immune Cells via the Production of Chemokines, Alarmins as Well as via Antigen Presentation

The finding that Fgf10 regulates the expression of immune genes in AT2 cells adds an additional understanding to the proposed immune function played by Fgf10. Is all the activity of Fgf10 on the immune cells carried out via Fgf10 action on AT2s, or does Fgf10 mediate this action via the LIFs? We also cannot exclude that Fgf10 could directly act on immune cells. Further studies are therefore necessary to define the role of AT2s and associated LIFs and immune cells in BPD.

While the role of Fgf10 in controlling inflammation is still unclear, the role of another Fgfr2b ligand, Fgf7, has been reported (Prince et al., 2001). In primary cultures of human airway epithelial cells, recombinant FGF7 negatively affected the expression of many interferon-induced genes (Prince et al., 2001). In addition, different biological responses have been reported depending on the timing of epithelial *Fgf7* overexpression using transgenic mice. Short time overexpression impacts positively the repair process following acute lung injury, while long time expression causes inflammation (Tichelaar et al., 2007).

### Deficiency of *Gm-csf* or Its Receptor Causes the Failure to Develop AMs Capable of Clearing Surfactant Proteins Made by AT2 Cells

In general, signals from the microenvironment regulate the maintenance and replacement of the AMs. One of these signals in particular, granulocyte-macrophage colony-stimulating factor (Gm-csf or Csf-2), is known to play an important role. Deficiency of Gm-csf or its receptor in mice mimics a human disease called pulmonary alveolar proteinosis, characterized by the accumulation of surfactant in the alveoli leading to impaired gas exchange (Trapnell et al., 2009). Gm-csf expression is impacted by the level of expression of Fgf10 in the mouse model of BPD previously described (see section “A Mouse Model of BPD Allows Studying Mechanistically the Significance of Reduction in *Fgf10* Expression”) (Chao et al., 2017). The physiological meaning of *Gm-Csf* expression changes in relation with Fgf10 expression should be further analyzed and contextualized in the inflammation process.

### Il33 Is a Cytokine Secreted by AT2s Potentially Regulated by Fgf10

AMs as well as other immune cells responsible for type 2 immunity (Symowski and Voehringer, 2017), such as innate lymphoid cells (specifically ILC2), basophils, eosinophils, and mast cells are present at low numbers at birth but increase during alveologenesis in mice (Starkey et al., 2019). During mouse lung development, a peak in the numbers of these cell types is observed when pulmonary remodeling is at its climax, strongly suggesting that type 2 immunity actively participates in the process (de Kleer et al., 2016). This peak is associated with a local Il33 increased concentration, a cytokine secreted by AT2s, and which is potentially regulated by Fgf10. It is proposed that Il33 acts on type 2 immune cells, leading to the activation and increased number of ILC2s, stimulation of AMs, as well as inhibition in the dendritic cells of the expression of IL12p35 (de Kleer et al., 2016). Active ILC2s control the repair process in the lung. In addition, as mechanical stress associated with breathing has been reported to increase Il33 secreted by AT2s, as well as by fibroblasts, it has been suggested that this mechanical stress-based Il33 release, especially in the context of the first breath at birth, could regulate alveologenesis in mice (Saluzzo et al., 2017). Indeed, it has been reported in the context of BPD that reduced FGF10 expression and/or increased inflammation are associated with impaired alveologenesis (Chao et al., 2017). Il33 is also increased in the hyperoxia mouse model.

### Lipopolysaccharides (LPS) Are Known to Be a Potent Activator of the Innate Immune System and Have Been Used in the Context of *in vitro* Lung Explant Culture

**FIGURE 1:**
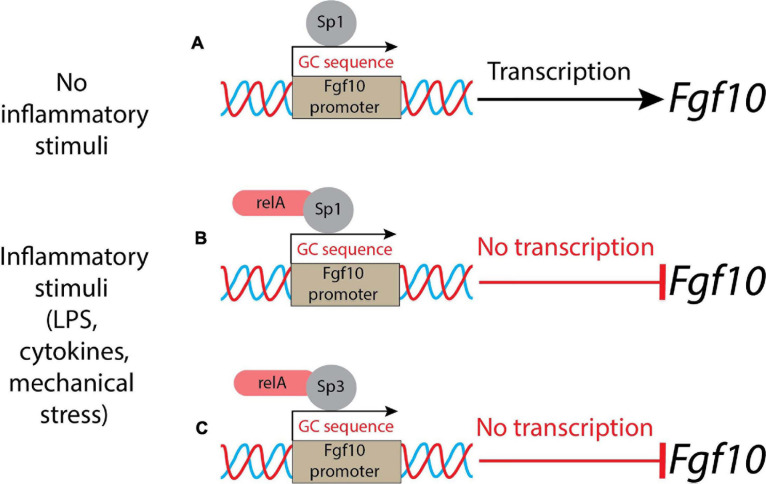
A schematic representation of control of *Fgf10* expression during lung morphogenesis. **(A)** In normal conditions (no inflammatory stimuli), Sp1 binds the Fgf10 promoter leading to the transcription of the growth factor; **(B)** in presence of inflammatory stimuli, the relA subunit of NF-κB translocates into the nucleus and binds Sp1, acting as a repressor; **(C)** alternatively, the relA subunit of NF-κB interacts physically with Sp3 and the complex competes with Sp1: the result is a suppression of *Fgf10* transcription.

Lipopolysaccharides activate the inflammation through Toll-like receptors 2 and 4 (Tlr2/4), which in turn leads to inhibition of *Fgf10* expression (Benjamin et al., 2007). The conditioned medium from LPS-induced lung explants reduces the branching process when added to WT, as well as in *Tlr4* null lung explants. This indicated that LPS triggered its action on the lung via inflammatory mediators. This *in vitro* assay also demonstrated the existence of a remarkable link between *Fgf10* expression and inflammation. The NF-κB pathway is the major signaling pathway active in BPD patients. In general, NF-κB is considered a transcriptional activator; however, in the case of the *Fgf10* gene, the activation of the NF-κB pathway is associated with *Fgf10* inhibition (Benjamin et al., 2010). As there are no binding sites for NF-κB in the *Fgf10* promoter, it has been proposed that this inhibition is indirect and could be mediated through the activation of a transcriptional repressor. *Fgf10* promoter analysis revealed several GC-rich regions which could be Sp protein binding sites. NF-κB interferes with Sp1 binding on the *Fgf10* promoter, but also on other promoters (e.g., *Bmp4, TgfβRIII*, *Col1A2*, and *Col2A1*) (Benjamin et al., 2010). Sp1 normally induces *Fgf10* expression and NF-κB plays the unusual role of a repressor (Figures 1A,B). Interestingly, the inhibition of *Fgf10* expression is dose-dependent: the higher is the inflammation, the less *Fgf10* is expressed. Furthermore, NF-κB mediates the binding of Sp3, another Sp family member, which acts as a transcriptional factor that stimulates or represses transcription similarly to Sp1, and both bind to similar GC-rich sequences (Carver et al., 2013) and localize into the nucleus. Upon inflammatory stimuli, relA, a subunit of NF-κB, translocates into the nucleus, where it interacts physically with Sp3 to help binding the *Fgf10* promoter, thereby inhibiting *Fgf10* gene expression (Carver et al., 2013) (Figure 1C). The Sp1-*Fgf10* promoter binding, responsible for the positive *Fgf10* expression, is overcome by the relA action on the availability of Sp3, with the shift in the balance to *Fgf10* repression: inflammatory stimuli determine the downregulation of *Fgf10*.

This process links the inflammatory response to the expression of a key gene orchestrating organogenesis. What is unclear so far is how changes in *Fgf10* expression could impact inflammation. Tlr2/4 are receptors for many small inflammatory molecules, including the alarmin high-mobility group box 1 (Hmgb1) (Wang et al., 2020). It has been recently reported that in lung bronchial epithelial cells, recombinant FGF10 prevents the translocation of this alarmin from the nucleus to the cytoplasm/extracellular compartment (Liu et al., 2020). This resulted in the prevention of the activation of the toll-like receptors, thereby blunting the inflammatory cascade. It is still unclear whether Fgf10 can directly act on immune cells, or if its effect on inflammation is mostly via its impact on AT2 cells.

## Link Between Inflammation and At2-LIF Interaction

### Fgf10 Is Expressed by Resident Mesenchymal Cells in the Lung

As mentioned before, Fgf10 is expressed by mesenchymal progenitors as well as by differentiated LIFs and acts in a paracrine/autocrine fashion via Fgfr1b and Fgfr2b (Al Alam et al., 2015). Previously, it was reported that Fgf10 is essential to promote the differentiation of mesenchymal progenitor cells toward the LIF lineage. LIFs are mesenchymal cells, displaying a high density of lipid droplets (Torday and Rehan, 2016). In the adult lung, LIFs and AT2s closely interact, and we propose that Fgf10 takes center stage in this interaction. Through their close interaction with AT2 cells, LIFs transfer triglycerides to AT2 cells to produce surfactants (Torday and Rehan, 2016). LIFs are also capable of eliciting the self-renewal of a special subpopulation of AT2s displaying stem cell capabilities (Barkauskas et al., 2013). This property has been explored and monitored, using the so-called “alveolosphere” assay, wherein AT2s and LIFs are isolated by flow cytometry and co-cultured in growth factor-reduced Matrigel for 2–3 weeks. During this time frame, organoids are formed which are made of Sftpc^Pos^-AT2 cells as well as Podoplanin^Pos^-AT1 cells (Barkauskas et al., 2013). Indeed, this reflects the property of AT2 stem cells which can proliferate to give rise to more AT2 stem cells, as well as differentiate toward the AT1 lineage. Therefore, it has been proposed that the LIF-AT2 interaction is critical for repair after injury (Nabhan et al., 2018; Zacharias et al., 2018). LIFs are quite poorly characterized and few markers are known to define LIFs: perilipin 2 (Plin2) (Ntokou et al., 2017), platelet derived growth factor receptor alpha (Pdgfrα) (Barkauskas et al., 2013), peroxisome proliferator-activated receptor gamma (Pparγ) (Rehan and Torday, 2012) and Fgf10 (Al Alam et al., 2015) in mice, and more recently, follistatin (FST) and APOE in humans (Travaglini et al., 2020).

Moreover, further characterization of the LIFs is required using approaches such as lineage tracing combined with single-cell RNA-seq data during development, homeostasis and repair after injury. These approaches will allow gathering important mechanistic information about potential cell surface markers capable of distinguishing between different sub-clusters of LIFs, as well as the signaling pathways involved in their differentiation and activation after injury. Our group has reported that in the postnatal mouse lung, *Fgf10* is only expressed by 30% of the LIFs, suggesting that LIFs are indeed heterogeneous (Al Alam et al., 2015). The difference between Fgf10^Pos^-LIFs and Fgf10^Neg^-LIFs from a biological point of view is still elusive. We propose that the Fgf10^Pos^-LIFs are likely the ones capable of sustaining the self-renewal of AT2 stem cells in organoid assays.

### The AT2-LIF Interaction *in vitro* Should be Investigated in Presence of Immune Cells

In the context of pneumonectomy in mice (which triggers a process of lung regeneration in the remaining lobes characterized by increased AT2 proliferation), it was reported that immune cells are essential for the expansion of AT2 cells (Lechner et al., 2017). The number of Cd115^Pos^ and Ccr2^Pos^ monocytes and M2-like macrophages increases at the peak of AT2 proliferation. Macrophages are recruited to the lung through the Ccl2/Ccr2 chemokine axis and are required for optimal AT2 proliferation and differentiation following pneumonectomy. It has been proposed that Il13, produced by type 2 innate lymphoid cells (ILC2), could promote lung regeneration, but its mode of action, direct or indirect on AT2s, is still unclear. These results support the concept that the manipulation of the immune cells could be a potential winning strategy to enhance the repair process (Lechner et al., 2017).

Mechanistically, candidate partners for the immune cells could be AT2s and/or LIFs. Use of *in vitro* organoid models bringing together AT2s, LIFs and immune cells should allow to decipher further the mechanistic aspects of such interactions. Like the LIFs, the AT2s are heterogeneous and contain a population of Wnt-responding cells displaying enhanced self-renewal capabilities *in vitro*, both in mice (Nabhan et al., 2018; Zacharias et al., 2018) and in humans (Travaglini et al., 2020). In addition, our group has recently identified a subpopulation of AT2 cells, distinct from these Wnt-responding cells, which are normally quiescent in homeostasis, but which can proliferate upon injury (Ahmadvand et al., 2021). Of note, this subpopulation of AT2 cells preferentially express some genes related to the immune system. AT2s in general are known to play immunomodulatory functions; they can produce cytokines (IL33, IL17) and modulate the recruitment of immune cells, such as neutrophils, to the site of infection (Kalb et al., 1991; de Kleer et al., 2016; Wosen et al., 2018). Mouse and human AT2s express major histocompatibility complex 2 (MHC II) proteins at the cell surface, therefore acting as antigen presenting cells to the CD4^Pos^ T-cells. Additionally, LIFs could also play a role in the modulation of the immune response. LIFs provide a source of neutral lipid (involved in surfactant production to alleviate the surface tension in the alveoli), which could be involved in mediating an immune response as well (Torday and Rehan, 2016; Duffney et al., 2018).

## Link Between Inflammation and FGF10 Signaling in Copd

### Chronic Obstructive Pulmonary Disease (COPD) Is the Third Major Cause of Lung Illness (and Death) in the World and Is Characterized by Chronic Lung Inflammation

Such an inflammatory response, overtime, leads to an obstruction of the conducting airways that is irreversible (Adeloye et al., 2015). Notably, the risk for COPD is also increased in humans displaying mutations in *FGF10* (Prince, 2018). Together with the branching defects which are the consequences of impaired lung development, constitutive *FGF10* insufficiency appears therefore to be associated with COPD. Macrophages and lymphocytes are the primary immune cells involved in the pathogenesis of this chronic disease (Nurwidya et al., 2016). Environmental causes are predominant in the pathogenesis of this disease. For instance, cigarette smoke and pollution mediated by particulate matter (PM) are guilty partners in COPD (Ling and van Eeden, 2009; Kelly and Fussell, 2011). Several studies have focused on PM (especially the ones equal or smaller to 2.5 μm than can easily reach the alveoli) that people breathe every day with long-term consequences (Ling and van Eeden, 2009).

### Positive Effect of Treatment With Recombinant FGF10 in a Mouse Model of Airway Lung Injury

Recently, the treatment with recombinant FGF10 gave a positive effect in a mouse model of airway lung injury triggered by PM exposure. The authors analyzed the effects on the survival and the outcome of the administration of exogenous FGF10, resulting in a strong improvement of the animals (Liu et al., 2020). Ameliorated inflammatory response in FGF10-treated mice was observed, with a decrease in cell infiltration and decrease in proinflammatory cytokine expression (Il6, Il8, Tnfα, and Pge2). One of the possible downstream players of Fgf10 has been identified as Hmgb1, a nuclear and cytoplasmic small protein; after treatment with exogenous FGF10, the level of Hmgb1 was decreased in BALF (bronchoalveolar lavage fluid).

**FIGURE 2:**
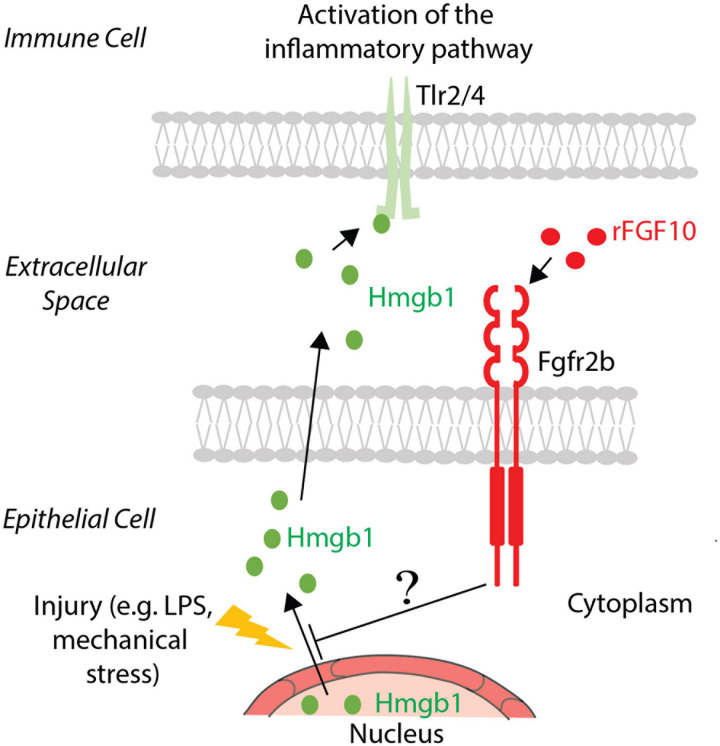
FGF10 effect on Hmgb1. In the case of injury, Hmgb1 shuttles from nucleus to the cytoplasm of the epithelial cells to be released in the extracellular space, where it could bind its receptors Tlr2/4 present on the immune cell surface, causing an inflammatory response. Exogenous FGF10 blocks the release in the extracellular space, acting on the shuttling of Hmgb1 from the nucleus to the cytoplasm. The involved mechanism and the mediators are still unknown.

As mentioned earlier, Hmgb1 is considered an alarmin/damage-associated molecular pattern protein and is ubiquitously expressed (Yang et al., 2020). It is secreted into the extracellular space in response to inflammation and, via its binding to Toll-like receptors, activates the inflammatory cytokines cascade (Liu et al., 2020). It was already reported that FGF10 inhibits the release of Hmgb1 from the nucleus to the extracellular domain, and therefore prevents its binding with Tlr2/4 and the activation of the signaling pathway. TLR4 was up-regulated like HMGB1 in human bronchial epithelial cells after PM-treatment. Administration of recombinant FGF10 reduced TLR4 expression and the shuttling of HMGB1 from the nucleus to the cytoplasm and extracellular space (Liu et al., 2020). The molecular mechanism underlying this inhibition is still not known. Altered expression of proteins involved in the trafficking or degradation of HMGB1 could potentially represent the missing links between FGF10 and inflammation (see Figure 2 for details). In addition, an increased number of inflammatory cells were detected, indicating that FGF10 is involved in the recruitment of immune cells to re-establish lung homeostasis (Liu et al., 2020).

### Increased Infiltration of Cd4^Pos^Cd25^Pos^Foxp3^Pos^ T Regulatory Cells in Fgf10 Overexpression Transgenic Mouse Model

The increase in inflammatory cell infiltration in the lungs of FGF10-treated mice in the context of PM exposure seems to occur also in other lung disease models. In a bleomycin-induced mouse model of lung fibrosis [a model for idiopathic pulmonary fibrosis (IPF)], alveolar epithelial Fgf10 overexpression using transgenic mice significantly increased the infiltration of Cd4^Pos^Cd25^Pos^Foxp3^Pos^ T regulatory cells during the inflammation phase (Gupte et al., 2009). How these cells are recruited by Fgf10 (i.e., directly or indirectly) is still unclear. Regulatory T-cells are usually involved in the tolerance to self-antigens, and regulate the other immune cells (Okeke and Uzonna, 2019). During this phase, many chemoattractant and signaling molecules are released, with an increase of immune cells in the tissues.

Other T-cell subpopulations could be involved in the homeostasis of the lung and in the modulation of the inflammatory response. In smokers with preserved lung function, an upregulation of γδ T-cell numbers has been reported (Urboniene et al., 2013; Johnson et al., 2020). This is in sharp contrast with COPD patients, where this number is lowered. These γδ T-cells are distinct from Cd4^Pos^Cd25^Pos^Foxp3^Pos^ T-regulatory cells. γδ T-cells are usually negative for Cd4 and Cd8 and express unique TCR receptors. γδ T-cells represent 8–20% of the total resident lymphocyte populations in the lung and are one of the major coordinators for the inflammation that is established in case of injury (Urboniene et al., 2013). It is therefore not surprising that the increased number of γδ T-cells is related only to the active smokers with a normal lung: this observation is compatible with a physiological response aimed at protecting or repairing the lungs from the injury caused by tobacco smoking.

## Inflammation and FGFs in the Lung and Other Organs

Apart from Fgf10, the role of other members of the Fgf family in regulating inflammation has also been reported. These Fgfs are expressed in organs where an active epithelial barrier function is required, such as the skin, gut, reproductive tract, and as already cited, the lung. These organs are characterized by the presence of cells able to produce and secrete Fgfs. Additionally, these organs also display the significant presence of specific γδ T-cells at the epithelial barrier (Cheng and Hu, 2017). Fgfs are also produced and secreted by different immune cells and act in a paracrine fashion to regulate the proliferation of epithelial cells, especially in cases where homeostasis needs to be re-established after injury.

### FGF7 and Inflammation

A well-reported example of this process occurs in the gastrointestinal tract of adult mice. In the gut, intraepithelial lymphocytes (IEL) secrete Fgf7, thereby enhancing proliferation and differentiation of intestinal epithelial cells (Visco et al., 2009). Fgf7 is an Fgfr2b ligand which can potentially act redundantly with Fgf10. Several inflammatory disease models were used to investigate the impact of recombinant FGF7 on inflammation: in a model of colitis in rats and in mice, administration of recombinant FGF7 after colitis induction decreased the damage to the intestine mucosa and was associated with diminished cell death (Zeeh et al., 1996; Egger et al., 1999). When dextran-sulfate-sodium (DSS) induced-colitis was performed in *Fgf7* KO mice, as well as in *TCRδ* KO mice, the severity of the gut damage between the two models was comparable, but importantly both KO lines displayed a higher susceptibility to the DSS-induced colitis compared to wild type mice used as controls. This study demonstrated the reparative role of Fgf7 in the intestinal mucosa, identifying also γδ intraepithelial T-lymphocytes as the cells producing and releasing the growth factor (Chen et al., 2002). Intriguingly, the damage in the intestinal barrier was slowly repaired, also in the absence of Fgf7 and TCRδ, suggesting the presence of compensatory mechanisms that may involve other growth factors.

### FGF2 and Inflammation

Other Fgfs such as Fgf2 also modulate inflammation. *Fgf2* expression was detected in macrophages and T-cells, both players in acute and chronic inflammation (Song et al., 2015; Shao et al., 2017). Several human studies revealed that FGF2 blood level is increased in Crohn’s disease and ulcerative colitis patients (Kanazawa et al., 2001) and that FGF2 could induce the expression of several proinflammatory genes in asthma (Tan et al., 2020). In inflammatory bowel disease (IBD) pathogenesis, the role of the inflammatory cytokines and the cells that produce them, T-helper1 and regulatory T-cells (specifically γδ T-cells), are well recognized. Administration of exogenous recombinant FGF2 alleviates DSS-induced colitis (Shao et al., 2017). Other studies dealing with lung diseases suggest that FGF2 may be associated with airway inflammation, bringing up the possibility of FGF2-targeted therapy. A recent review summarized the role of FGF2 as an immunomodulatory factor in COPD and severe asthma (Tan et al., 2020). In lung diseases, the inflammatory component is often responsible for the exacerbation of the pathology. Against this background, Song et al. (2015) investigated the relationship between Fgf2 and Il17 in the context of a DSS-colitis-induced model. In this model, a high number of infiltrating cells, specifically γδ T-cells, was observed. These cells produced both Il17 and Fgf2 which interact through nuclear factor NF-kappa-B activator 1 (Act1). Indeed, Act1 connects Fgf2 and Il17 signaling, allowing a synergistic action between these two pathways. In this study, dysregulated microbiota driven by the Tgfβ1-Fgf2 axis cooperates with Il17 to promote the repair of the damaged intestinal epithelial barrier, and Act1 bridges the direct signaling cross-talk between Fgf2 and Il17 for the cooperative effect. Such a synergistic effect was also tested in the context of rheumatoid arthritis (RA), an autoimmune disease (Zhang et al., 2019). In their collagen-induced arthritis (CIA) mouse model, ectopic expression of *Fgf2* caused a severe form of the disease, but in the *Il17* KO mice, these effects triggered by Fgf2 were blunted, thereby demonstrating that Fgf2 requires Il17 to mediate its effects. IL17 is also involved in the pathogenesis of different lung diseases such as COPD and IPF (Bozinovski et al., 2015). Innate and adaptive T-cells, as well as epithelial cells in the lung, have been proposed as a source for IL17. In lung fibrosis, IL17 seems to be primarily produced by γδ T-cells and the recruitment of those cells has been shown as a positive outcome. Recombinant FGF10 may recruit T-regulatory cells at the site of inflammation, similarly to Fgf2 in the DSS-colitis model, and thereby stimulate the release of IL17, to drive repair and homeostasis.

## Use of FGF10 as a Therapy in Inflammatory Disease

### FGF10 and Dry Eye Disease

FGF10 modulates inflammation and as such, its administration in the context of disease could be beneficial. For example, local FGF10 administration via drops directly into the eye could represent a possible therapeutic solution to dry eye disease (Zheng et al., 2015). In a rabbit model of dry eye disease, such treatment resulted in the improvement of the general health of the eye. Indeed, the inflammatory process, which is visualized by the presence of necrotic areas, is clearly decreased in the context of FGF10 treatment. FGF10 increases the proliferation of the corneal epithelium and apoptosis is resolved (Zheng et al., 2015). In this context, the FGF10 effect on inflammation could be indirect; it probably ended the signals that sustained the apoptosis of the cells, a process that triggered and/or potentiated the inflammatory process. In addition, increasing the proliferation of the remaining healthy cells could also overcome the inflammatory signals, leading to the resolution of the damages (Zheng et al., 2015). Against this background, it is critical to mention that the alarmin Hmgb1 could be released from necrotic cells. As previously described, Hmgb1, after binding Tlr4, elicits the activation of a major inflammatory pathway, the NF-κB pathway.

### FGF10 and Acute Kidney Injury

In the kidney, ischemia/reperfusion (I/R) causes acute kidney injury (AKI) and results in necrotic areas that could release inflammatory signals (Kezić et al., 2017). The impact of recombinant FGF10 pre-treatment in a mouse model of I/R, that mimics AKI, has been investigated. It was shown that FGF10 prevented the decrease of nuclear Hmgb1, implying that FGF10 stops the shuttling from the nucleus to the cytoplasm of the alarmin, thereby preventing the consequent binding with, and activation of, Tlr2/4, which thereby leads to the inhibition of the inflammatory cascade (Kezić et al., 2017). In agreement with this model of action, inhibition in pro-inflammatory cytokine production, like Il6 and Tnfα, was reported, revealing a similarity to what happens in the particulate matter-driven inflammation in the mouse lung.

### FGF10 and Spinal Cord Injury

Tlr4 expression and the NF-κB pathway are also involved in spinal cord injury (SCI) (Chen et al., 2017). In this traumatic injury, microglia/macrophages and neurons produce Fgf10. In a mouse model of SCI, FGF10 treatment depressed the triggered inflammatory factors (Tnfα and Il6) and the NF-κB signaling pathway, and increased phosphorylation of the Pi3K/Akt signaling pathway. Notably, blockade of the Pi3K/Akt signaling pathway was not sufficient to impair the anti-inflammatory action of FGF10 (Chen et al., 2017). Once again, FGF10 appears to prevent inflammation through the inhibition of the NF-κB pathway and the associated inflammatory cytokines. Further studies investigated how recombinant FGF10 administration could affect the consequences of injury to the central nervous system (CNS) (Li et al., 2016). It was demonstrated that FGF10 treatment inhibited the activation and proliferation of microglia/macrophages by inhibiting the Tlr4/NF-κB pathway, thereby attenuating the production and release of pro-inflammatory cytokines after SCI.

Moreover, increased expression of *Fgf10* from the neurons and microglia/macrophages after acute SCI has been reported. We propose that such increase could be an endogenous self-protective response, supporting the idea that Fgf10 ameliorates the inflammatory process at sites of injury. The involvement of Tlr4, one of the receptors for Hmgb1, opens the question whether the activity through this receptor could be modulated by Fgf10. *In vitro* assays identified the Tlr4/NF-κB pathway as the target of FGF10 anti-inflammatory effect; when LPS-induced BV2 cells (microglia cells) were pre-treated with FGF10, they displayed reduction in the expression of Trl4 and attenuation of NF-κB activation. FGF10 significantly impacts also on p65 (relA) nuclear translocation.

### FGF10 and Chronic Inflammatory Skin Disease

The anti-inflammatory effect of FGF10 is not universal. It is known, for instance, that that while FGF10 plays an important role during organogenesis of the skin (Suzuki et al., 2000), in the context of psoriasis, a chronic inflammatory skin disease characterized by excessive proliferation of the keratinocytes, *Fgf10* together with *Fgf7* and their common receptor *Fgfr2b* were overexpressed (Kovacs et al., 2005). Psoriasis is characterized by the presence of a lymphocyte infiltrate that correlates with the level of *Fgf10* expression. In the corresponded animal model, blocking antibodies against Fgf10 ameliorate the inflammatory response, with a significant reduction (Xia et al., 2014). Taken together, evidence suggests that Fgf10 contributes to inflammation in the skin.

## What’s Next?

### Better Characterization of the AT2 Cells

The reciprocal interaction between Fgf10 and inflammation both during organogenesis and the repair process after injury has been an important research topic. From a mechanistic aspect, emerging evidence points to the contribution of immune cells to the proliferation and differentiation of epithelial progenitor cells. Such activity of the immune cells qualifies them to be an integral part of the niche which supports the self-renewal and differentiation of these epithelial progenitor cells. In the context of the lung, AT2 cells are one of the most studied epithelial progenitor cells. These cells interact with mesenchymal cells as well as immune cells. An important aspect of these basic interactions is that each of these cell types represents a heterogeneous population. It is therefore extremely difficult to identify a specific role for each player. For the AT2 cells, our recently published work suggests that a subpopulation of quiescent and relatively undifferentiated AT2 cells, positive for the immune marker PD-L1, could be a major partner for the immune cells (Ahmadvand et al., 2021). In addition, Fgf10 signaling via Fgfr2b in AT2 cells appears to control the expression of immune markers (Chao et al., 2017). It is not clear from the reported study whether this regulation occurs in mature AT2 cells and/or in quiescent PD-L1^Pos^-AT2 cells. One additional complication concerns the mesenchymal cells *per se*. LIFs, positive for Pdgfrα, have been reported to constitute the niche for AT2 cells in the alveolosphere organoid model (Barkauskas et al., 2013).

However, the LIFs are heterogenous, and more work needs to be done to identify the subset of LIFs responsible for the niche activity. As an example of such heterogeneity, only 30% of the LIFs express Fgf10 postnatally. It will be interesting to explore further, using the alveolosphere model, the functional difference between a Fgf10^Pos^-LIF and a Fgf10^Neg^-LIF. Fgf10 secreted by the Fgf10^Pos^-LIF can either act in an autocrine fashion on the LIFs themselves to maintain their differentiation status, or act in a paracrine fashion on AT2 cells. The expected activity of Fgf10 signaling in the differentiated AT2 cells is still unclear. It may involve survival, proliferation, as well as differentiation (Gupte et al., 2009; Jones et al., 2019). The role of Fgf10 signaling on PD-L1^Pos^-AT2 quiescent cells is equally unclear. However, in the context of pneumonectomy (PNX), these cells proliferate and differentiate toward mature AT2s. They also display an upregulation of *Fgfr2b* and *Etv5* expression in PNX versus Sham controls, suggesting increased Fgf signaling. In a SftpcCre-Tomato (Tom) mouse model, in which a tamoxifen Cre recombinase is under the control of the promoter of the surfactant protein C (Sftpc), it is possible to identify the AT2 cells using Tom expression/fluorescence. The positive cells display a different expression level of Tom. The isolation of these cells leads to the identification of two different populations, Tom^hi^ and Tom^low^, based on the level of Tomato expression. Further analysis of these cells led to the discovery of Tom^Low^ cells displaying the expression of immune markers. It will be important to decipher the role of Fgf signaling on the expression of these markers in this AT2 sub-population using specific driver lines targeting these cells. This can be achieved using Dre/Rox and Cre/LoxP technology to generate two complementary driver lines, one under the control of the CD274 promoter and the other one under the control of the *Sftpc* promoter. Finally, the immune cells themselves are a big black box. The macrophages in the lung, for example, originate from at least three different sources (Tan and Krasnow, 2016). The subpopulation of resident or circulating macrophages interacting with AT2 or LIF subpopulations is so far unclear, and more work will have to be done in the context of development and repair after injury to tackle this important issue.

### Better Characterization of the Interaction of AT2s With Immune Cells

Recently, many studies have focused on the identification of the specific signature of the type of cell responsible for the regeneration process of the lung in case of injury (e.g., LPS and bleomycin). Three studies converge on the identification of a transitional state, that characterizes AT2 cell differentiation into AT1, in order to re-establish tissue homeostasis. The authors named this new progenitor state with different names, including pre-alveolar type 1 transitional state (PATS), alveolar differentiation intermediate state (ADI) and damage associated transient progenitors (DATP) (Choi et al., 2020; Kobayashi et al., 2020; Strunz et al., 2020). Verheyden and Sun (2020) summarized and compared the features of these apparently different populations, highlighting how they are probably a picture of the same transitional state. PATS, ADI, and DATP are characterized by high expression of Krt8 and activation of TP53 genes and by a senescence signature. In Choi’s paper, the authors, using as a model the bleomycin-treated mice, confirmed interleukin 1β (Il-1β) as a possible inducer of the transitional state. The gene signature of primed AT2 cells corresponded to an activated immune state, similar to what is reported in Ahmadvand’s paper more recently (Ahmadvand et al., 2021). In Choi’s paper, they identified IM as the source of Il-1β. Il-1β is secreted by IM after bleomycin treatment and acts on the AT2s that express the receptor Il1r1 leading to the acquisition of the transitional status. Il-1β administration on *ex vivo* AT2-derived organoids isolated from Sftpc+ lineage tracing mice leads to the activation of AT2s and pushes them into the transitional state, in which the cells display a specific expression profile, with an upregulation of the genes regulated by TP53, together with high expression of keratin 8 (Krt8) and claudin 4 (Cldn4). The fact that the induction of the transitional state comes from a cytokine secreted by macrophages is strong evidence of the impact of the immune system in tissue homeostasis. The inflammatory signals seem to have a prominent role in the regeneration process, and it is possible that they drive the regulation of the process itself, at least in part. In case of inflammation, the recruited circulating monocytes that arrive at the site show many IM features (Gibbings et al., 2017). At this point, it is overly reductive to consider only immune cells as players in the canonical inflammatory response. Macrophages in the lung already show heterogeneity and plasticity, and it appears that they could display different roles. Further research will be required to identify more subsets responsible for the homeostasis or repair processes. In the same way that AT2 cells are heterogeneous, it is more likely that macrophages, both alveolar and interstitial, with the additional contribution of the recruited-monocytes, could be characterized by different sets of genes, based on the type of environment they face: either healthy or compromised (such as after injury by chemical compound or by bacteria/virus).

### Better Characterization of the Macrophages

Many different surface markers are used to identify the different macrophage subpopulations, including recruited-monocytes, but they are not yet sufficient to delineate the different subsets. However, among them, Cx3cr1 and Ccr2 appear to be good candidates to follow the fate and to explore the dynamics of circulating-monocytes. Recently, it has been shown that cells positive for Ccr2 in the subtissular niche of the lung are present in IMs which display variable Cx3cr1 expression (Chakarov et al., 2019). Using these two surface receptors, it is possible to identify macrophages derived from circulating monocytes: Ccr2^hi^Cx3cr1^low^ are usually considered to be classical or pro-inflammatory macrophages and Ccr2^low^Cx3cr1^hi^ are alternative or patrolling monocytes. The dual reporter system Cx3cr1^*GFP/*+^Ccr2^*RFP/*+^, which allows to study the fate of these peculiar cells, will be instrumental to follow the monocytes recruitment in the tissues in presence of damage/injury. The identification at the same time of the change in expression of these two markers led to the possibility to discriminate different macrophage subpopulations based on the expression level of these receptors. This system has led to the discovery of the dynamic reprogramming of monocytes in different tissues, like the liver and the brain (Dal-Secco et al., 2015; Faustino et al., 2019). It is still unclear what induced the switch of expression in the surface markers and what happened to these cells when they reached their target tissues. Ccr2 seems to guide their recruitment following the gradient of Ccl2 (Ccr2 counterpart). Upon arrival in their target organ, they are proposed to undergo reprogramming quickly. Cx3cr1 expression characterized the cells involved in wound healing, but how and through what mechanism remains to be investigated. Moreover, due to the high heterogeneity of the macrophage population and the high variation in surface marker expression, in a context of regeneration, it could be interesting to adopt a depletion/deficiency approach; several models are available, and they are well summarized in a recent review (Hua et al., 2018). A depletion model, such as CD169-DNT, where a diphtheria toxin inducible system enclosing a fusion product of the human DNT receptor under control of the CD169 sialoadhesin gene promoter (Miyake et al., 2007), could clarify the role of this specific subset of macrophages. Similarly, additional studies on the different populations of lung macrophages, including AM and IM and recruited-monocytes, are required to specifically identify unique markers to target for transient depletion or genetic studies.

## Discussion

**FIGURE 3:**
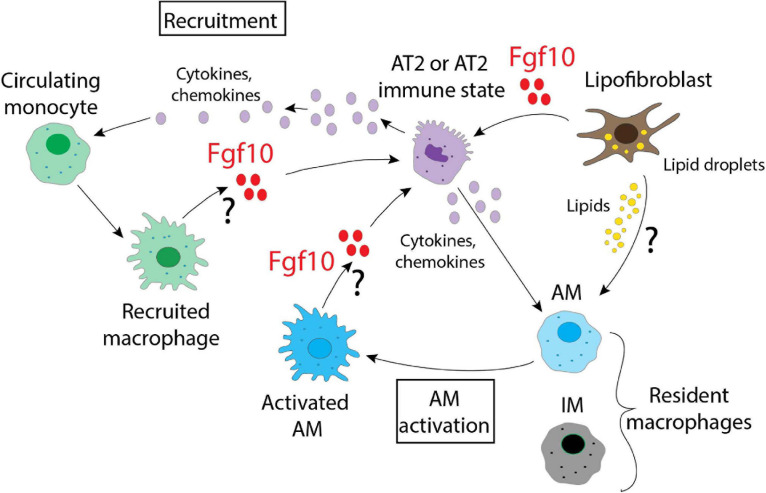
A schematic representation of the possible interactions between different alveolar and immune cells in the lung. Fgf10, produced by lipofibroblasts, recruited macrophages and/or activated AMs, targets AT2 cells. Fgf10 can also orchestrate the production/release of chemokines and cytokines. Lipofibroblasts, via the secretion of lipids, can also activate the AMs.

What emerges from this picture is the plasticity of the immune cells and their ability to adapt and follow new signals from the microenvironment which, in the lung, are likely generated by the heterogenous populations of LIFs and/or AT2s. It is therefore likely that multiple cell populations respond to stress as represented in Figure 3. Further studies are necessary to identify what guides the immune cells to sites of injury and the signals that later regulate them. From the data already published, Fgf10 is surely one of the most interesting candidates due to the evidence of its connection to several immune molecules, and it would therefore be important to decipher its role in detail by identifying the cells responsible for its production and release. This should be done not only in a context of injury/repair, but also during lung organogenesis, where Fgf10 controls the expression of many genes linked to the immune system. The possibility to use different mouse models that express different levels of Fgf10, together with the Cx3cr1/Ccr2 dual reporter system that identifies dynamic subpopulations of IM, could provide interesting clues about the complex interactions that are relevant in the cross-talk occurring in the microenvironment. In summary, Fgf10 is a key player during organogenesis, repair after injury, as well as in the inflammation, and its use, by clinicians, as a therapy to repair a damaged epithelial barrier may be more popular if beneficial effects on the inflammatory process are also robustly established.

## Author Contributions

MM and SB wrote the review and made the illustration. CC and SB edited the review. All authors contributed to the article and approved the submitted version.

## Conflict of Interest

The authors declare that the research was conducted in the absence of any commercial or financial relationships that could be construed as a potential conflict of interest.
